# Raddeanin A down‐regulates androgen receptor and its splice variants in prostate cancer

**DOI:** 10.1111/jcmm.14267

**Published:** 2019-03-23

**Authors:** Hongyan Xia, Cheng Hu, Shanshan Bai, Jing Lyu, Bryan Y. Zhang, Xianghui Yu, Yang Zhan, Lijing Zhao, Yan Dong

**Affiliations:** ^1^ National Engineering Laboratory for AIDS Vaccine School of Life Sciences Jilin University Changchun China; ^2^ Department of Structural and Cellular Biology Tulane University School of Medicine Tulane Cancer Center New Orleans Louisiana; ^3^ Lusher Charter School New Orleans Louisiana; ^4^ School of Nursing Jilin University Changchun China

**Keywords:** androgen receptor, castration‐resistant prostate cancer, raddeanin A, splice variant

## Abstract

Castration‐resistant progression of prostate cancer is a major cause of prostate cancer mortality, and increased expression and activity of the full‐length and the splice variants of androgen receptor (AR) have been indicated to drive castration resistance. Consequently, there is an urgent need to develop agents that can target both the full‐length and the splice variants of AR for more effective treatment of prostate cancer. In the present study, we showed that raddeanin A (RA), an oleanane‐type triterpenoid saponin, suppresses the transcriptional activities of both the full‐length and the splice variants of AR. This is attributable to their decreased expression as a result of RA induction of proteasome‐mediated degradation and inhibition of the transcription of the AR gene. We further showed the potential of using RA to enhance the growth inhibitory efficacy of docetaxel, the first‐line chemotherapy for prostate cancer. This study identifies RA as a new agent to target both the full‐length and the splice variants of AR and provides a rationale for further developing RA for prostate cancer treatment.

## 1 INTRODUCTION

Prostate cancer is the second‐leading cause of cancer‐related mortality in men in Western countries. The growth and survival of prostate cancer cells rely on androgen receptor (AR), which is activated by androgens (reviewed in Ref. 1). Consequently, androgen deprivation therapy via surgical or medical castration remains the standard of remedy for locally advanced or metastatic disease. However, within 2‐3 years after androgen deprivation therapy, the majority of the patients progresses to castration‐resistant prostate cancer (CRPC), which is the major cause of prostate cancer mortality (reviewed in Ref. 1). AR activity remains active in CRPC, and increased expression of AR and its splice variants, AR variants (AR‐Vs), which lack the ligand‐binding domain, is an important mechanism of AR reactivation in CRPC (reviewed in Refs. 1, 2).

The full‐length AR (AR‐FL) consists of three major domains, the N‐terminal transactivation domain followed by the DNA‐binding domain and the C‐terminal ligand‐binding domain. The ligand‐binding domain is connected to the DNA‐binding domain by a flexible hinge region (reviewed in Refs. 3, 4). When activated by androgens, AR is translocated to the nucleus, forms a homodimer, and binds to regulatory regions of target genes to regulate gene expression (reviewed in Ref. 5). In contrast, AR‐Vs lack the ligand‐binding domain, but the majority contains intact N‐terminal transactivation domain and DNA‐binding domain and thus possesses constitutive transcriptional activity.^6^, ^7^, ^8^, ^9^, ^10^, ^11^, ^12^ High expression of AR‐Vs, specifically, AR‐V7, AR^v567es^ and AR‐V9, has been associated with poor prognosis and short survival of CRPC patients.^8^, ^9^, ^13^, ^14^, ^15^, ^16^, ^17^ Therefore, development of drugs that can target both AR‐FL and AR‐Vs has been an active area of research in combatting CRPC.

Raddeanin A (RA) is an oleanane‐type triterpenoid saponin extracted from the root of *Anemone raddeana* Regel, a traditional Chinese medicinal herb used to treat rheumatism and arthritis in ancient China.^18^ Preclinical studies have indicated the antitumour activity of RA against gastric cancer, colorectal cancer, breast cancer, liver cancer, choriocarcinoma, glioblastoma and osteosarcoma.^19^, ^20^, ^21^, ^22^, ^23^, ^24^, ^25^, ^26^, ^27^, ^28^ In the present study, we sought to investigate the anticancer effect of RA in prostate cancer. We found that it could suppress the activity of both AR‐FL and AR‐Vs to inhibit the growth of prostate cancer cells at an in vivo achievable concentration.^29^, ^30^

## 2 MATERIALS AND METHODS

### 2.1 Cell lines, reagents and SRB assay

DU145, 22Rv1 and PC‐3 cells were purchased from the American Type Culture Collection. C4‐2 and C4‐2B cells were obtained from Dr Shahriar Koochekpour at Roswell Park Cancer Institute, and LNCaP95 cells were provided by Dr Alan Meeker at Johns Hopkins University. All cell lines are cultured in RPMI1640 supplemented with penicillin, streptomycin, and 10% foetal bovine serum. All cell lines were within 20 passages, authenticated, and tested negative for mycoplasma contamination. RA was purchased from Yuanye Biological (Shanghai, China). For RA treatment in the presence or absence of R1881, a synthetic androgen, the cells were cultured in medium containing charcoal‐stripped serum. Otherwise, the cells were cultured with normal serum during the course of the experiments. Cell growth was assessed by the sulforhodamine (SRB) assay as described,^31^ and the SRB assay was performed at least three times in six replicates.

### 2.2 Western blot analysis

Western blot analysis was performed with a standard protocol. Briefly, ~20 μg of protein samples were resolved over 10%‐15% SDS/PAGE and transferred to a polyvinylidene fluoride membrane. After blocking in blocking buffer (5% non‐fat dry milk, 10 mmol/L Tris, pH 7.5, 10 mmol/L NaCl and 0.1% Tween 20), the membrane was incubated with a primary antibody overnight at 4°C, followed by incubation with a fluorescent‐labelled secondary antibody for 1 hour at room temperature. Membranes were scanned and analysed using an Odyssey^®^ Infrared scanner (LI‐COR Bioscience). The following antibodies were used: anti‐AR (Catalog No. 5153, Cell Signaling Technology, USA) and anti‐GAPDH (Catalog No. BA2913, Boster Biological Technology, USA). The Western blot analysis was performed at least three times, and AR levels were normalized by GAPDH levels.

### 2.3 DNA transfection and reporter gene assay

Transfection was performed with the use of the Turbofect reagent (Thermo) according to the manufacturer's protocol. Three luciferase reporter plasmids were used: ARR3‐luc (driven by three repeats of the probasin androgen‐responsive element^32^), UBE2C‐luc (driven by a minimal promoter and three repeats of an AR‐V‐specific promoter element of the ubiquitin conjugating enzyme E2C [UBE2C] gene^33^), and pGL4‐ARpro1.7 (driven by a 1.7 kb fragment of the 5′‐flanking region of the human AR gene^34^). To ensure even transfection efficiency, we conducted the transfection in bulk, and the cells were split into 24‐well plates 6 hours later for treatment with RA. The reporter gene assay was performed at least three times in triplicate.

### 2.4 Quantitative reverse transcription‐PCR

The Quantitative reverse transcription‐PCR (qRT‐PCR) analysis was performed as described.^35^ The TaqMan^®^ PCR primers for AR‐FL, AR‐V7, 36B4, prostate specific antigen (PSA) and UBE2C were purchased from Sangon Biotech (Shanghai, China). The primer sequences are: AR‐FL (forward: 5′‐GTACAGCCAGTGTGTCCGAA‐3′, reverse: 5′‐TTGGTGAGCTGGTAGAAGCG‐3′), AR‐V7 (forward: 5′‐AAAAGAGCCGCTGAAGGGAA‐3′, reverse: 5′‐GCCAACCCGGAATTTTTCTCC‐3′), PSA (forward: 5′‐CTCAGGCCAGGTGATGACTC‐3′, reverse: 5′‐GTCCAGCACACAGCATGAAC‐3′), UBE2C (forward: 5′‐TTCCCCAGTGGCTACCCTTA‐3′, reverse: 5′‐CAGGGCAGACCACTTTTCCT‐3′) and 36B4 (forward: 5′‐CGACCTGGAAGTCCAACTAC‐3′, reverse: 5′‐ATCTGCTGCATCTGCTTG‐3′). The qRT‐PCR analysis was performed at least three times in triplicate, and AR, PSA and UBE2C levels were normalized by 36B4 levels.

### 2.5 Statistical analysis

The Student's two‐tailed *t* test was used to determine the mean differences between two groups. *P *<* *0.05 is considered significant. Data are presented as mean ± SD from at least three independent experiments.

## 3 RESULTS

### 3.1 RA inhibits the growth of prostate cancer cells

**Figure 1:**
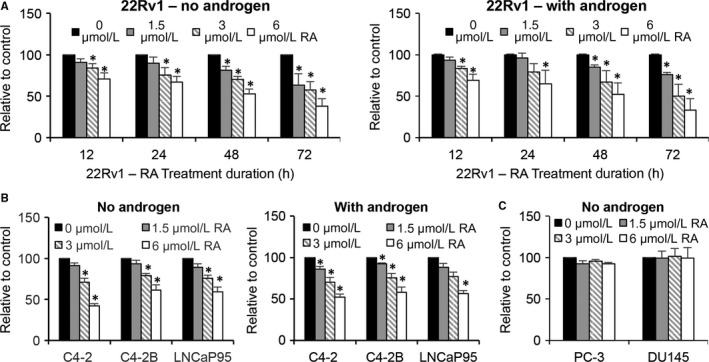
Raddeanin A (RA) inhibits the growth of prostate cancer cells. A, Sulforhodamine (SRB) assay shows RA inhibiting the growth of 22Rv1 cells in a time‐ and dose‐dependent manner either in the absence or presence of 1 nmol/L R1881, a synthetic androgen. B, SRB assay shows a dose‐dependent inhibition of the growth of C4‐2, C4‐2B, and LNCaP95 cells by RA at the 24‐h time point. C, SRB assay shows no inhibition of PC‐3 or DU145 cell growth by RA. *P <* *0.05 from the control group

We first assessed the effect of RA on the growth of CRPC cells by the SRB assay. The assay was conducted in the presence or absence of 1 nmol/L R1881, a synthetic androgen, for the AR‐expressing 22Rv1, C4‐2, C4‐2B and LNCaP95 cells and in androgen‐deprived condition for the AR‐null PC‐3 and DU145 cells. The doses of RA that we tested were from 0 to 6 μmol/L. This was because RA can reach a maximum plasma concentration of ~4.5 μmol/L in rats after a single intraperitoneal administration at 0.75 mg/kg.^29^ As presented in Figure 1A, B, RA inhibited the growth of all AR‐positive cells in a dose‐ and/or time‐dependent manner. The inhibition appears to be independent of androgen. In contrast, no growth inhibition was observed in the AR‐null cells (Figure 1C). These results suggested that RA‐induced growth inhibition in CRPC cells might be AR dependent but androgen independent.

### 3.2 RA suppresses AR signalling

**Figure 2:**
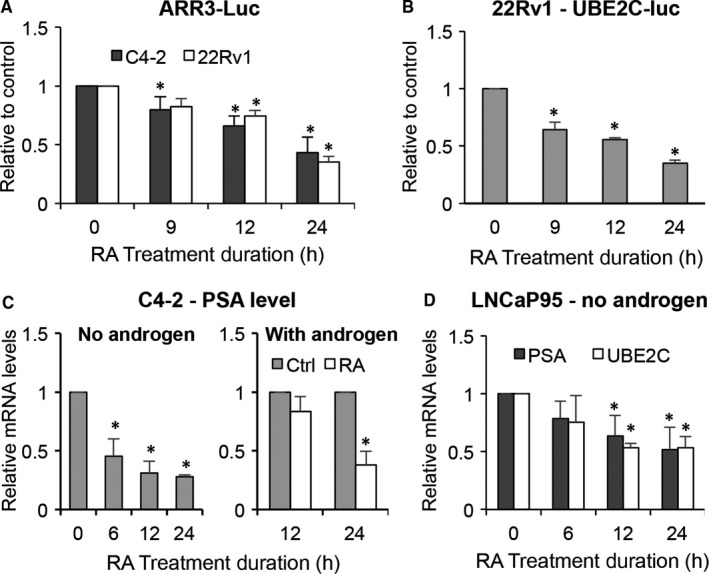
Raddeanin A (RA) down‐regulates AR‐FL and AR‐V transactivation. A and B, Luciferase assay shows RA inhibiting AR‐FL and AR‐V transcriptional activity. Cells transfected with the ARR3‐luc or UBE2C‐luc construct were treated with 3 μmol/L RA. C and D, qRT‐PCR analysis shows RA decreasing the levels of PSA and UBE2C mRNA. Cells cultured in the absence or presence of 1 nmol/L R1881, were treated with 3 μmol/L RA. *P <* *0.05 from the control group

We therefore investigated the effect of RA on AR transcriptional activity by reporter gene assay. We first transfected C4‐2 and 22Rv1 cells with the ARR3‐luc luciferase construct, which contains three tandem repeats of androgen‐response elements, and treated the cells with 3 μmol/L RA. As shown in Figure 2A, RA treatment led to a reduced luciferase activity as early as 9 hours after treatment, and the reduction became more pronounced with time. The ARR3‐luc construct can be regulated by both AR‐FL and AR‐Vs. However, as C4‐2 cells do not express AR‐Vs, the reduced luciferase activity in these cells provided clear evidence for the ability of RA to inhibit AR‐FL *trans*‐activating activity. To specifically assess the effect of RA on AR‐V transcriptional activity, we transfected 22Rv1 cells, which express both AR‐FL and AR‐Vs, with the UBE2C‐luc construct in which the luciferase gene is driven by an AR‐V‐specific promoter element of the UBE2C gene.^33^ Similar to the effect on AR‐FL, RA caused a time‐dependent inhibition of AR‐V *trans*‐activating activity (Figure 2B). Consistently, both basal and androgen‐induced expression of the canonical AR target PSA and the expression of the AR‐V‐specific target UBE2C were significantly down‐regulated by RA (Figure 2C and D). Importantly, the down‐regulation was evident prior to significant changes in cell growth, indicating that the effect was unlikely secondary to growth inhibition. Collectively, these data support the ability of RA to inhibit AR‐FL and AR‐V transactivation.

### 3.3 RA down‐regulates AR protein

**Figure 3:**
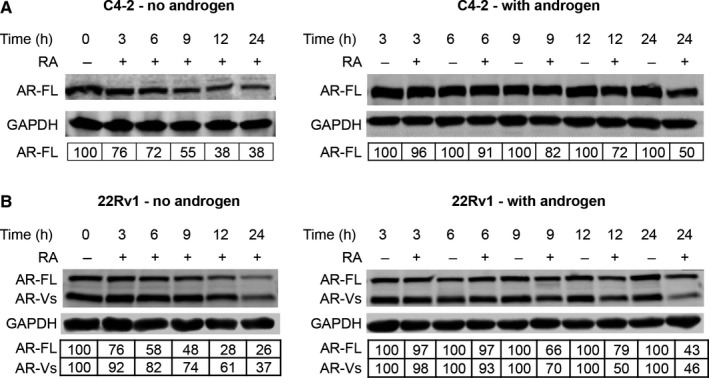
Raddeanin A (RA) down‐regulates AR‐FL and AR‐V proteins. C4‐2 (A) and 22Rv1 cells (B) cultured in androgen‐deprived condition were treated with 3 μmol/L RA in the presence or absence of R1881. The Western blot analysis was performed at least three times, and AR levels were normalized by GAPDH levels. The numbers in the tables denote relative normalized intensities compared to the control value of 100

To understand the mechanism by which RA inhibits AR transactivation, we examined AR protein levels after RA treatment. C4‐2 and 22Rv1 cells were treated with RA either in the presence or absence of androgen. RA down‐regulated both AR‐FL and AR‐V proteins (Figure 3). The effect on AR‐FL appeared to be more significant in androgen‐deprived condition, likely due to the lower stability of the AR‐FL protein in the absence of androgen. Taken together, the data suggested that RA inhibition of AR transactivation could be mediated through down‐regulating AR protein levels.

### 3.4 RA induces proteasome degradation of AR

**Figure 4:**
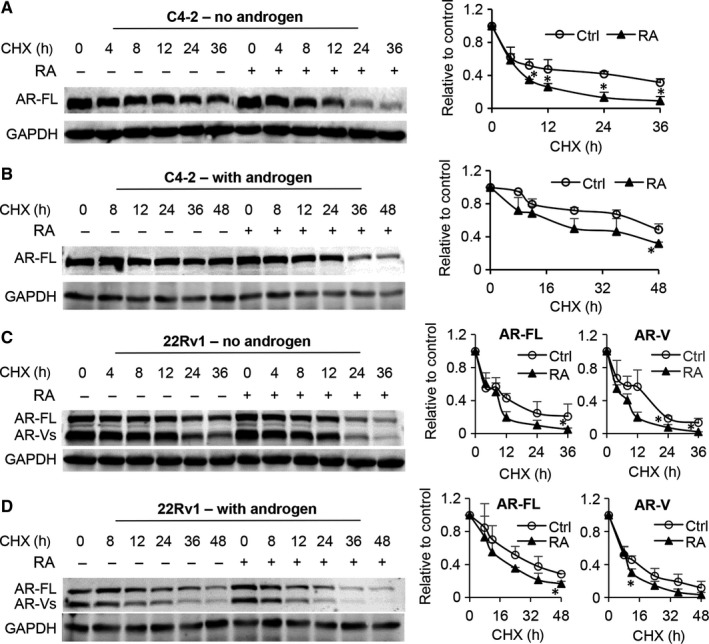
Raddeanin A (RA) reduces the stability of AR‐FL and AR‐V proteins. C4‐2 and 22Rv1 cells were treated with 10 μg/mL cycloheximide (CHX) with or without 3 μmol/L RA for the indicated time in the absence (A and C) or presence (B and D) of R1881. The right panels are the quantification of AR protein levels. Ctrl, control. *P <* *0.05 from the control group

**Figure 5:**
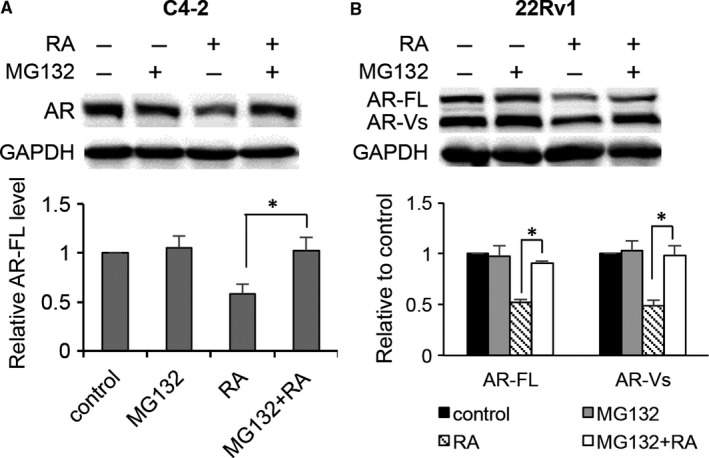
Raddeanin A (RA) down‐regulation of AR‐FL and AR‐V proteins involves the proteasome pathway. Western blotting showing MG132 attenuating RA down‐regulation of AR‐FL and AR‐V proteins in C4‐2 (A) and 22Rv1 cells (B). Cells were treated with 3 μmol/L RA with or without 10 μg/mL MG132 in androgen‐deprived condition. *P <* *0.05

To determine whether the decrease in AR proteins was due to increased protein degradation, we treated C4‐2 and 22Rv1 cells with cycloheximide to stop protein synthesis and monitored the decay rates of AR‐FL and AR‐V proteins in response to RA treatment either in the presence or absence of androgen. As shown in Figure 4, in both conditions, RA increased the decay rates of AR‐FL and AR‐V proteins. It has been described that proteasome‐mediated pathway is the main machinery regulating AR protein degradation.^36^, ^37^, ^38^ To determine the role of proteasome in RA‐induced AR degradation, we assessed the effect of the proteasome inhibitor MG132 on RA down‐regulation of AR proteins. As shown in Figure 5, the addition of MG132 greatly attenuated RA down‐regulation of AR, restoring the levels of AR‐FL and AR‐V proteins to almost the control level. Collectively, these data indicated that inducing proteasome‐mediated degradation of AR‐FL and AR‐V proteins is a mechanism by which RA decreases their expression.

### 3.5 RA suppresses the transcription of the AR gene

**Figure 6:**
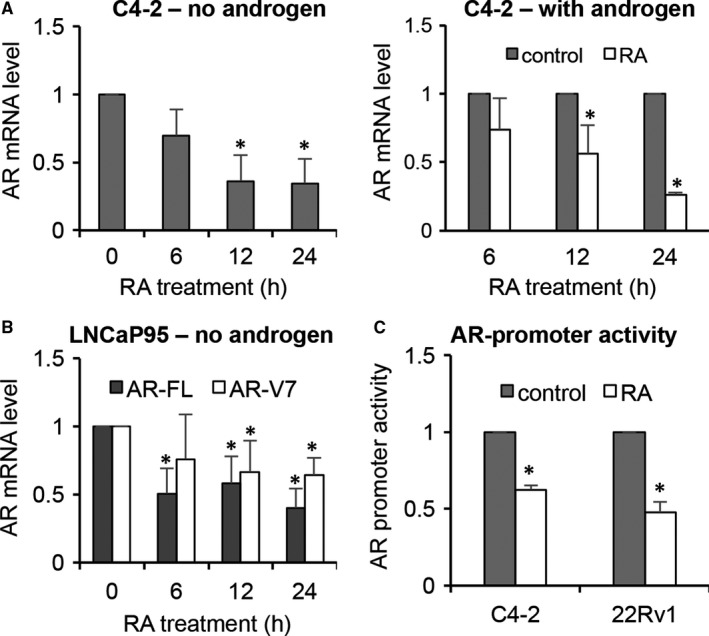
Raddeanin A (RA) inhibits the transcription of the AR gene. A and B, qRT‐PCR analysis showing RA, at 3 μmol/L, decreases AR‐FL and AR‐V mRNA levels in a time‐dependent manner in the presence or absence of 1 nmol/L R1881. C, Luciferase assay showing RA inhibition of the activity of a 1.7 kb proximal AR promoter. Cells transfected with the pGL4‐ARpro1.7 construct were treated with 3 μmol/L RA for 12 h. *P <* *0.05 from the control group

To investigate whether RA could modulate AR at the RNA level in addition to inducing AR protein degradation, we measured AR‐FL and AR‐V7 mRNA levels by qRT‐PCR. Interestingly, RA reduced the levels of both AR‐FL and ‐V7 mRNA (Figure 6A and B). Moreover, RA treatment led to a significant inhibition of the activity of a 1.7 kb proximal AR promoter (Figure 6C). Collectively, these findings indicated that RA down‐regulates AR at both transcriptional and post‐translational levels.

### 3.6 RA enhances the growth inhibitory efficacy of docetaxel

**Figure 7:**
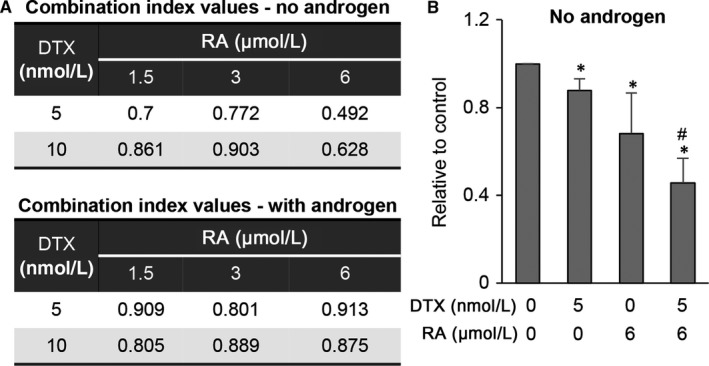
Raddeanin A (RA) enhances docetaxel efficacy in prostate cancer cells. A, Calculation of combination‐index values shows RA and docetaxel (DTX) synergistically inhibit the growth of 22Rv1 cells in the presence or absence of R1881. B, Graph shows the combination with the best synergistic effect. 22Rv1 cells cultured in androgen‐deprived condition were treated with 6 μmol/L RA with or without 5 nmol/L DTX for 48 h, and cell growth was assessed by the SRB assay. *P <* *0.05 from the control group. ^#^ *P *<* *0.05 from the single‐agent treatment groups

As the first‐line chemotherapy for patients with metastatic CRPC, docetaxel has been shown to inhibit nuclear translocation and transcriptional activity of AR‐FL.^39^, ^40^, ^41^, ^42^, ^43^, ^44^, ^45^ However, AR‐Vs, especially AR‐V7, are resistant to docetaxel modulation, and this has been proposed to be a mechanism of docetaxel resistance.^43^, ^45^, ^46^ Because of the ability of RA to down‐regulate AR‐V expression and activity as well as its ability to inhibit AR‐FL through a different mechanism from docetaxel, we hypothesized that RA may enhance the efficacy of docetaxel in CRPC. To test this hypothesis, we assessed the growth of 22Rv1 cells in response to treatment with RA and docetaxel in combination and calculated the combination index values, which delineate the interactions between the two drugs. A combination index value of <1, 1, or >1 denotes synergism, additivity, or antagonism, respectively. All the combinations produced a combination index value of less than 1, suggesting a synergy between RA and docetaxel in inhibiting cell growth (Figure 7A). The synergy was more pronounced in androgen‐deprived condition compared to the androgen‐present condition. Shown in Figure 7B is the combination that produced the best synergy. Cell growth was more significantly inhibited by the combination treatment than by single‐agent treatments. These data provided support for the potential of using RA to enhance docetaxel efficacy in CRPC.

## 4 DISCUSSION

The present study represents the first to characterize the activity of RA in prostate cancer, particularly CRPC. We showed that RA inhibits the growth of CRPC cells in a dose‐ and time‐dependent manner. While independent of androgen, the inhibition appears to be dependent on AR, as AR‐null cells are not impacted by RA treatment. Mechanistically, we showed that RA suppresses the transcriptional activities of AR‐FL and AR‐Vs, and this is attributable to decreased expression of AR‐FL and AR‐Vs. The decreased expression is a result of RA induction of proteasome‐mediated degradation of AR‐FL and AR‐V proteins and reduction of the transcription of the AR gene.

The AR‐FL protein is known to suppress the transcription of the AR gene, producing a negative feedback on the expression of AR mRNA.^47^ Consequently, RA‐mediated decrease of AR protein would be expected to lead to upregulated AR mRNA expression. Nevertheless, our results showed that RA could inhibit the transcription of the AR gene and reduce AR‐FL and AR‐V mRNA levels, indicating that RA could turn on a mechanism counteracting AR negative auto‐regulation. This is significant because it could lead to a sustained down‐regulation of AR‐FL and AR‐Vs, and further study is needed to identify the mechanism.

While supporting the anticancer activity of RA that has been indicated in other cancer types,^19^, ^20^, ^21^, ^22^, ^23^, ^24^, ^25^, ^26^, ^27^, ^28^ our findings unveil a tissue‐specific effect of RA in prostate cancer. That is to target AR‐FL and AR‐Vs. Increased expression of the full‐length and splice variants of AR has been indicated to be an important mechanism of resistance to traditional androgen deprivation therapy and the new androgen deprivation drugs abiraterone and enzalutamide (reviewed in Refs. 1, 2). However, none of the anti‐androgens currently used in clinics can target AR directly to reduce its availability. In addition to RA, several other compounds have been shown pre‐clinically to reduce the levels of AR‐FL and AR‐Vs.^34^, ^48^, ^49^, ^50^, ^51^, ^52^, ^53^, ^54^, ^55^, ^56^, ^57^, ^58^ These compounds may serve as an effective antidote to overcoming resistance to androgen deprivation therapy for treatment of CRPC.

These compounds may also have the potential to improve the efficacy of the first‐line chemotherapy for prostate cancer, docetaxel. Androgen‐induced translocation of AR‐FL to the nucleus, which is required for the transcriptional activity of AR‐FL, has been reported to use a microtubule‐facilitated pathway.^39^, ^40^, ^43^, ^46^ By stabilizing microtubules, docetaxel has been shown to attenuate AR‐FL nuclear import.^39^, ^40^, ^41^, ^42^, ^43^ On the other hand, the nuclear localization of AR‐Vs, especially AR‐V7, is independent of microtubule and thus insensitive to docetaxel inhibition.^43^, ^46^ As a result, AR‐V expression has been proposed to be a mechanism of docetaxel resistance.^43^, ^46^ Here, we showed that, by down‐regulating AR‐FL and AR‐V expression and activities, RA enhances the growth inhibitory efficacy of docetaxel in CRPC cells. RA has a low bioavailability if administered orally.^28^, ^30^ With a single oral administration of 1.5 mg/kg RA to mice and 2 mg/kg RA to rats, the maximum plasma concentration can only reach 12‐13 nmol/L, and the majority of RA is distributed to the intestinal tract, particularly the colon and caecum.^28^, ^30^ However, a maximum plasma concentration of ~30 or 3 μmol/L can be reached after intravenous or intraperitoneal administration of rats with 0.75 mg/kg RA, and the concentration can sustain in the μmol/L range for 6‐8 hours.^29^, ^30^ Therefore, determining the best route of RA administration and the best sequences of the combination treatment is needed. Taken together, the findings from the current study provide a rationale for further developing RA or its analogue for intervention of CRPC.

## CONFLICT OF INTEREST

The authors confirm that there are no conflict of interest.

## AUTHOR CONTRIBUTIONS

HX and CH performed the research. HX, CH, SB, JL, BYZ, LZ, XY, YZ and YD contributed to research design and data analysis and interpretation. HX, YZ and YD wrote the paper, BYZ revised the paper and all the authors approved the submitted manuscript.
